# N-Net: Lesion region segmentations using the generalized hybrid dilated convolutions for polyps in colonoscopy images

**DOI:** 10.3389/fbioe.2022.963590

**Published:** 2022-10-07

**Authors:** Rongsheng Cui, Runzhuo Yang, Feng Liu, Chunqian Cai

**Affiliations:** ^1^ College of Electronic Information and Optical Engineering, Nankai University, Tianjin, China; ^2^ Tianjin Key Laboratory of Optoelectronic Sensor and Sensing Network Technology, Nankai University, Tianjin, China; ^3^ First Teaching Hospital of Tianjin University of Traditional Chinese Medicine, Tianjin, China; ^4^ National Clinical Research Center for Chinese Medicine Acupuncture and Moxibustion, Tianjin, China

**Keywords:** N-shape deep neural network, generalized hybrid dilated convolution, colonoscopy, colorectal polyp identification, lesion region segmentation, deep learning

## Abstract

Colorectal cancer is the cancer with the second highest and the third highest incidence rates for the female and the male, respectively. Colorectal polyps are potential prognostic indicators of colorectal cancer, and colonoscopy is the gold standard for the biopsy and the removal of colorectal polyps. In this scenario, one of the main concerns is to ensure the accuracy of lesion region identifications. However, the missing rate of polyps through manual observations in colonoscopy can reach 14%–30%. In this paper, we focus on the identifications of polyps in clinical colonoscopy images and propose a new N-shaped deep neural network (N-Net) structure to conduct the lesion region segmentations. The encoder-decoder framework is adopted in the N-Net structure and the DenseNet modules are implemented in the encoding path of the network. Moreover, we innovatively propose the strategy to design the generalized hybrid dilated convolution (GHDC), which enables flexible dilated rates and convolutional kernel sizes, to facilitate the transmission of the multi-scale information with the respective fields expanded. Based on the strategy of GHDC designing, we design four GHDC blocks to connect the encoding and the decoding paths. Through the experiments on two publicly available datasets on polyp segmentations of colonoscopy images: the Kvasir-SEG dataset and the CVC-ClinicDB dataset, the rationality and superiority of the proposed GHDC blocks and the proposed N-Net are verified. Through the comparative studies with the state-of-the-art methods, such as TransU-Net, DeepLabV3+ and CA-Net, we show that even with a small amount of network parameters, the N-Net outperforms with the Dice of 94.45%, the average symmetric surface distance (ASSD) of 0.38 pix and the mean intersection-over-union (mIoU) of 89.80% on the Kvasir-SEG dataset, and with the Dice of 97.03%, the ASSD of 0.16 pix and the mIoU of 94.35% on the CVC-ClinicDB dataset.

## 1 Introduction

Colorectal cancer is the cancer with the second and the third highest incidence rates for the female and the male, respectively. Early diagnosis has a huge impact on the survival from colorectal cancer (Torre et al., 2015). Colorectal polyps are potential prognostic indicators of the colorectal cancer, and colonoscopy is the gold standard for the biopsy and the removal of colorectal polyps (van Toledo et al., 2022). Research shows that nearly half of the individuals taking the colonoscopy at the age of 50 are found to be suffered from colorectal polyps (Lima Pereira et al., 2020). This incidence rate even increases with the age (Rundle et al., 2008). The accurate identification of colorectal polyp lesion regions plays a preliminary role in the medical treatment of colorectal cancers (Ren et al., 2019; Qadir et al., 2020; Tan et al., 2020). However, several studies indicate that the missing rate of polyps through manual observations in colonoscopy can reach 14%–30%, depending on the types and the sizes of the polyps (Van Rijn et al., 2006). Thus, the development of accurate colorectal polyp segmentation methods is critical.

With the rapid development of computer and information techniques, computer-aided diagnosis methods have been used in polyp segmentation tasks. However, the techniques of computer-aided polyp segmentations is still immature, especially in the cases that some complex and uncontrolled environmental factors exist. For example, existing computer-aided diagnosis methods can not effectively deal with factors that can affect the accuracy of polyp segmentation, such as intraluminal folds and variations of the polyp textures and locations. In (Sasmal et al., 2018), the principal component pursuit (PCP) technique was used in colorectal polyp segmentations. However, the segmentation performance got worse in low-light situations. In (Ganz et al., 2012), the shape-UCM methods were used in colorectal polyp segmentations, but the polyps with heterogeneous shapes could not be extracted.

Later, some early machine learning methods were developed for colorectal polyp segmentations. However, these methods consumed a lot of computer memory and relied excessively on handcrafted features, thus were not robust enough. For example, the machine learning based methods in (Tajbakhsh et al., 2016; Yu et al., 2017) got constrained segmentation performance for polyps in the presence of high luminance or intestinal residues.

In the past few years, the deep learning technology has been adopted in the segmentations of medical images. Particularly, Olaf proposed the U-shape artificial neural network (U-Net) using an encoding-decoding structure (Ronneberger et al., 2015). In the U-Net, multiple encoding and decoding modules were included in a symmetrical framework. In addition, the skip connections were added between the encoding and decoding paths to enable multi-scale information transmissions. The encoder-decoder structure of the U-Net has now become the most commonly used network structure in medical image segmentations.

More recently, some network structures based on the U-Net were proposed for the medical image segmentations and showed outstanding performances (Zhou Z. et al., 2018; Chen et al., 2018; Oktay et al., 2018; Huang et al., 2020; Chen et al., 2021). In particular, the densely connected convolutional neural network (DenseNet) strengthened the feature propagation and alleviated the gradient-vanishing problem, with the correct training convergences and the good feature extraction performances ensured (Huang et al., 2017; Wang et al., 2021). Moreover, in (Alom et al., 2018; Gu et al., 2021; Zhang and Yang, 2021), the attention mechanisms were also proposed recently, greatly improving the precision of medical image segmentations. Additionally, in order to further reduce the training time of the accelerate the convergence of the network training, the transfer learning was adopted by scholars (Shao et al., 2015; He et al., 2020; Rozo et al., 2022).

In addition to the above works, researches showed that the fine-grained image features could be better captured by expanding the receptive fields in the multi-scale information transmissions. In (Zhou L. et al., 2018), L. Zhou *et al* connected the encoding and decoding paths with dilated convolutions, where the receptive fields were expanded. However, the gridding effects could be introduced in this case. In order to solve this problem, P. Wang et al proposed the hybrid dilated convolution (HDC) (Wang et al., 2018). The HDC was established with cascaded dilated convolutions and has been adopted for semantic segmentations (Fu et al., 2019; Cheng et al., 2020; Liu et al., 2020; Ma et al., 2022). For example, J. Liu et al implemented an HDC based algorithm in the detection of retinal pigment epithelium defective cells (Liu et al., 2020). However, the mathematical model of the strategy of HDC designing was not much described. In addition, the HDC architecture required all the convolutional kernel sizes to be equal, with its implementation flexibility constrained.

**FIGURE 1:**
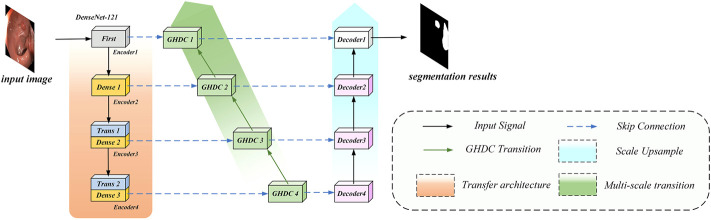
The overview of our proposed N-Net.

In order to overcome the shortcomings of the current methods and optimize the colorectal polyp lesion region segmentation performance using colonoscopy images, we propose a novel N-shaped artificial neural network (N-Net) structure. It should be specially noted that although this work focuses on the colorectal segmentation task, the N-Net structure can be used generally for image segmentations. The N-Net structure is briefly described in Figure 1. Compared with state-of-the-art methods through experiments on two public colonoscopy datasets for polyp segmentations, the proposed method achieves the best segmentation performance in the metrics of Dice, average symmetric surface distance (ASSD) and mean intersection-over-union (mIoU). The main contributions of this paper are as follows:

1) We propose a novel N-shaped artificial neural network (N-Net) structure to conduct the lesion region segmentations of polyps in colonoscopy images. The proposed N-Net is designed based on the encoding-decoding framework. Within the proposed structure, the multi-scale information can flow between the encoding and decoding paths. The pretrained DenseNet modules based on the ImageNet are implemented in the encoding path of the N-Net to ensure the fast training convergence and good feature extraction performance of the entire network structure.
2) To expand the receptive fields and facilitate the multi-scale information transmission between the encoding and decoding paths, we propose a strategy to design the generalized hybrid dilated convolution (GHDC). Compared with the existing works related to the dilated convolutions, the GHDC is established with a more flexible strategy to design cascaded dilated convolutional layers.
3) Based on the strategy of GHDC designing, four GHDC blocks are designed to connect the encoding path and the decoding path. With experiments on two public available datasets: the Kvasir-SEG dataset and the CVC-ClinicDB dataset, we show that the GHDC blocks outperform the HDC in (Wang et al., 2018). Moreover, comparative studies shows that the proposed N-Net, even with a small amount network parameters, outperforms the state-of-the-art methods including TransU-Net, DeepLabV3+ and CA-Net.

## 2 Methods

### 2.1 The structure of the N-Net

**FIGURE 2:**
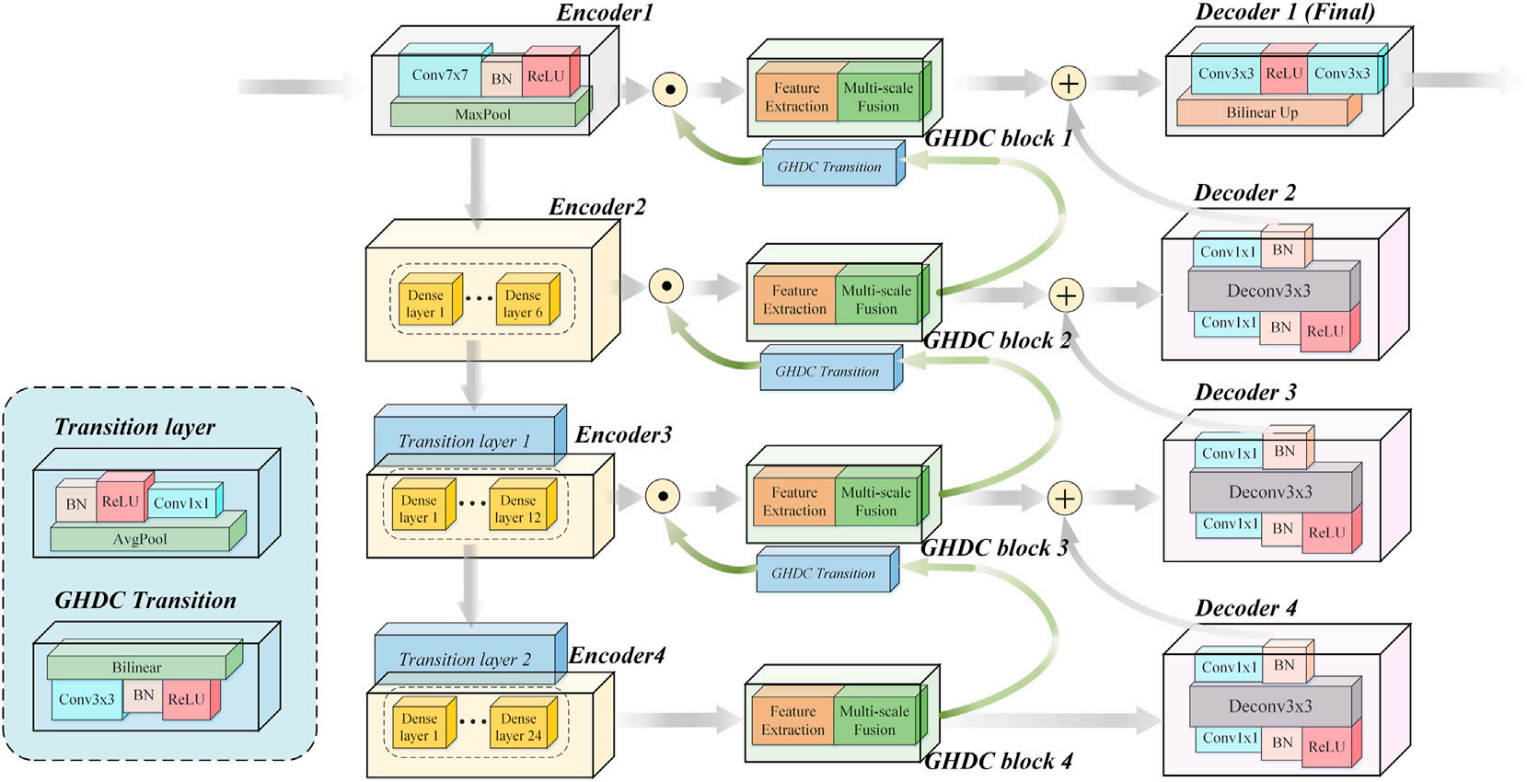
The detailed structure of our proposed N-Net.

In this paper, motivated by the U-Net encoder-decoder framework and the DenseNet modules, we propose a novel N-shaped architecture. As shown in Figure 2, the proposed N-Net structure contains four stages both in the encoding path on the left side and the decoding path on the right side. In addition, the GHDC blocks are added to connect the encoding and the decoding paths.

In contrast to the U-Net structure, a “Conv (7 × 7)-BN-ReLU” operation (i.e. convolution with the kernel size of 7 × 7 followed by a batch normalization and a ReLU) and an “Maxpool” operation (maximum pooling with the kernel size of 7 × 7 and the stride of 2) are implemented as the first stage of the encoding path. With the idea of transfer learning, the three encoding modules in the encoding path are designed based on the three dense blocks in the pre-trained DenseNet-121 from the ImageNet, respectively. Each dense block is composed by certain dense layers and a transition layer. The pre-trained dense layers in the three dense blocks and the first two transition layers are directly transferred. The number of dense layers in the three dense blocks are 6, 12 and 24, respectively. The number of output channels from the four encoders are 64, 256, 512 and 1,024, respectively. The feature map size from the Encoder 1 is 224 × 224 and is halved after the processing of each encoder.

The decoding path in the proposed N-Net is composed with four decoding modules. Each of the decoding modules contains a “Conv (1 × 1)-BN-ReLU-TransposeConv (3 × 3)-BN-Conv (1 × 1)” procedure (i.e. 3 steps: the first and third steps consist of a convolution with 1 × 1 kernel and a batch normalization; the second step consists of a transpose convolution with 3 × 3 kernel and a batch normalization). The Decoder one contains two “Conv (3 × 3)” operations and a bilinear interpolation operation.

The encoding and decoding paths in the N-Net structure are connected with the proposed GHDC blocks, which are designed based on the novel strategy of GHDC designing in this paper. For *i* = 1, 2, 3, the outputs of the Encoder *i* and the GHDC block *i* + 1 are concatenated as the input of the GHDC block *i*. Besides, the outputs of the GHDC block *i* and the Decoder *i* + 1 are point-wise added as the input to the Decoder *i*.

With the connections between the encoding path and the decoding path using the GHDC blocks, the multi-scale features are deeply exploited with expanded respective fields. The detailed strategy of GHDC designing and the GHDC blocks are provided in the remainder of this section.

### 2.2 The strategy of generalized hybrid dilated convolution designing

In order to achieve sufficient multi-scale information transmissions, while expanding the respective fields without the gridding effects, we establish a more general and simplified strategy to design the cascaded dilated convolutions. In contrast to the HDC, flexible dilation rates and convolutional kernel sizes are enabled in the designed operation. Which is referred to as the generalized hybrid dilated convolution (GHDC) in this paper.

**FIGURE 3:**
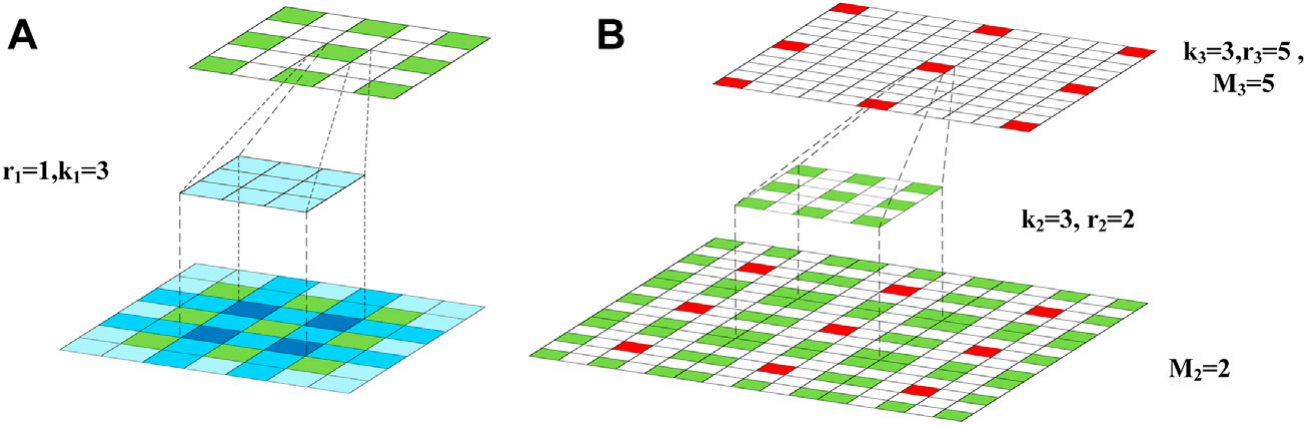
The cascaded dilated convolutional layers: **(A)** The operation of the first dilated convolutional layer; **(B)** the operation of the second dilated convolutional layer.

To explain the strategy of GHDC designing, we first provide a simple example to design the first three layers in a cascade of dilated convolutions in Figure 3. The convolutional kernel sizes for the three layers are denoted as *k*_1_, *k*_2_ and *k*_3_, respectively. The dilation rates used in the three layers are denoted as *r*_1_, *r*_2_ and *r*_3_, respectively.

In Figure 3A, the feature map into the first layer, the dilated convolutional kernel of the first layer and the feature map into the second layer are shown from down to up, respectively. In Figure 3B, the feature map into the second layer, the dilated convolutional kernel of the second layer and the feature map into the third layer are shown from down to up, respectively. In the feature map into the first layer, the darker color suggests that the corresponding pixels are used for more times during the convolution. In the feature maps into the second and third layers, the maximum distances between the neighbored pixels, which are sampled in the dilated convolutions, are denoted as *M*_2_ and *M*_3_, respectively. According to Figure 3, *M*_2_ and *k*_1_ can be used to observe the coverage of the convolutional kernel in the first layer.

In the proposed strategy of GHDC designing, a key point is to ensure that all of the pixels in the feature map into the first layer are utilized in the operations in order to avoid the gridding effect.

To meet this constraint, the parameters in the third and second layers are decided sequentially. For example, if *M*_3_, *k*_2_ and *r*_2_ takes the value of 5, 3 and 2, respectively, then *M*_2_ becomes 2. In this scenario, by taking *k*_1_ = 3 and *r*_1_ = 1, it can be guaranteed that no holes exist during the cascaded convolutions.

To be more general, we observe the dilated convolution operation on the arbitrary *lth* (*l* > 0) layer in a dilated convolution cascade. In this scenario, it can be found that the sampling positions in this operation are influenced by the parameters of both the *lth* and (*l* + 1)^*th*^ layers. Let us define *M*_*l*+1_ as the maximum distance between neighbored sampled positions horizontally or vertically on the (*l* + 1)^*th*^ (*l* > 0) layer, and define *r*_*l*_ and *k*_*l*_ to be the dilation rate and the convolutional kernel sizes on the *lth* layer, respectively. Then we can estimate *M*_*l*_ by the sliding of the dilated convolutional kernel on an *xOy* coordinate system. Due to the symmetry, there is no harm to simplify the problem by just observing the movement of the convolutional kernel on the *x*-axis.

If we consider *k*_*l*+1_ pixels separated by the distance of *M*_*l*+1_ on the feature map into the (*l* + 1)^*th*^ layer, then the possible pixels covered by the convolutional kernel can be represented by: {(*m*_*l*,*i*_ *r*_*l*_ + *n*_*l*,*i*_ *M*_*l*+1_, 0)}, where $m_{l,i}\in \left\{0,\pm 1,\ldots ,\pm \frac{k_{l}-1}{2}\right\}$ and $n_{l,i}\in \left\{0,\pm 1,\ldots ,\pm \frac{k_{l+1}-1}{2}\right\}$ stand for the coefficients to determine the location of *ith* pixel covered by the convolutional kernel. In other words, if *M*_*l*+1_, *k*_*l*+1_, *r*_*l*_, *k*_*l*_ are determined, the parameter *M*_*l*_ can be derived.

Therefore, in the proposed GHDC model, the design of a dilated convolution cascade is done in a recursive manner. For the *lth* (*l* > 0) layer, we define *D*_*l*_ = {*d*_*l*,*i*_}, *i* = 1, … , *k*_*l*_ *k*_*l*+1_–1 to be a non-decreasing sequence. Then given *M*_*l*+1_ and *k*_*l*+1_, *r*_*l*_ and *k*_*l*_ (*l* > 1) are determined with the following constraints:

$$d_{l,i}=\|\left(m_{l,i+1}r_{l}+n_{l,i+1}M_{l+1}\right)-\left(m_{l,i}r_{l}+n_{l,i}M_{l+1}\right)\|,$$ (1)

where ‖ ⋅‖ stands for the l-2 norm operation, $m_{l,i}\in \left\{0,\pm 1,\ldots ,\pm \frac{k_{l}-1}{2}\right\}$ and $n_{l,i}\in \left\{0,\pm 1,\ldots ,\pm \frac{k_{l+1}-1}{2}\right\}$ . Then *M*_*l*_, which is used to design the parameters of *k*_*l*−1_ and *r*_*l*−1_, can be determined as follows:

$$M_{l}=Max\|d_{l,i}-d_{l,i-1}\|\text{ and }M_{2}\leq k_{1}.$$ (2)

In addition, it is preferred that the receptive field in the first dilated convolution layer, which can be calculated using:

$$F=1+\underset{l}{\sum }r_{l}\left(k_{l}-1\right),$$ (3)

can cover the input feature map to the entire cascaded dilated convolutions.

For instance, if the kernel sizes of three are used in the cascaded dilated convolutions, we can get the following expression using Eqs 1, 2:

$$M_{l}=\left\{\begin{array}{c} r_{l},0<M_{l+1}<r_{l}\;\\ \max\left\{r_{l},M_{l+1}-2r_{l}\right\},M_{l+1}>2r_{l}\; \end{array}\right.$$ (4)

By recursively solving Eq. 4, we can get a cascade of convolutional layers, which is consistent with the HDC.

### 2.3 The GHDC blocks

**FIGURE 4:**
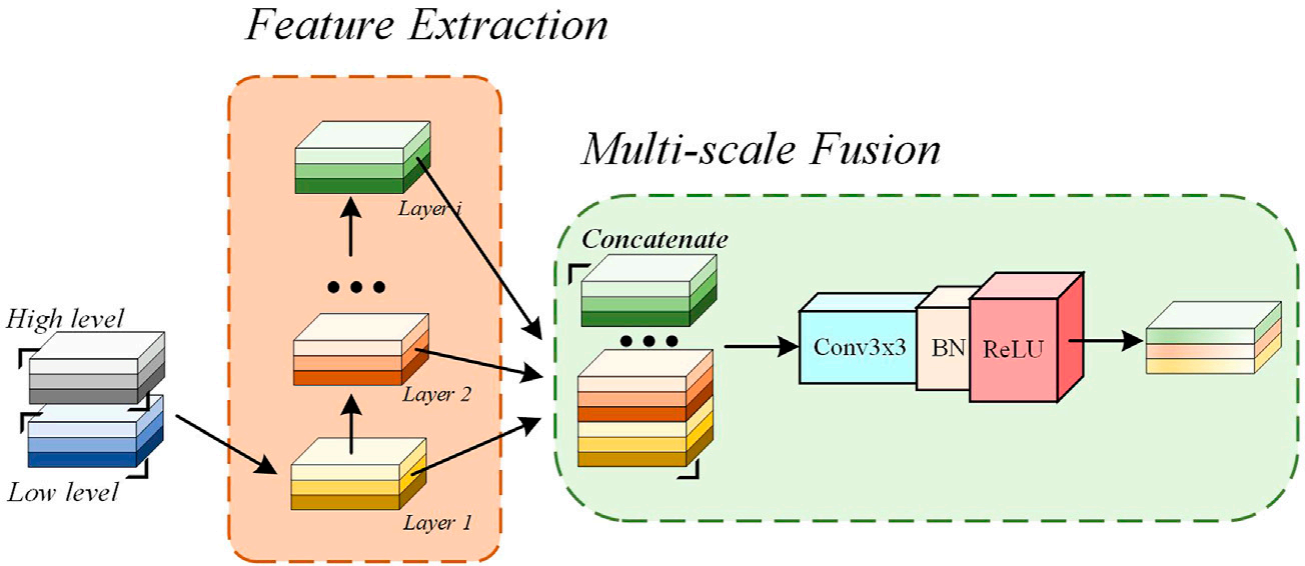
The Overview of the Proposed GHDC block.

In the proposed N-Net, the GHDCs are conducted with four GHDC blocks between the encoding and decoding paths. As shown in Figure 4, a GHDC block generally consists of a feature extraction module and a multi-scale fusion module. The feature extraction module is established with parallel groups of cascaded dilated convolutional layers, which are designed according to the strategy of GHDC designing. In the multi-scale fusion module, the results from the groups of cascaded dilated convolutional layers are concatenated and then processed using a “Conv (3 × 3)-BN-ReLU” operation to keep the output size of each GHDC block consistent with its input. In addition, to make sure that the feature map dimensions from the GHDC block *i* + 1 are consistent with that from the Encoder *i* (*i* = 1, 2, 3), a GHDC transition layer, consisting of a bi-linear interpolation and a “Conv (3 × 3)-BN-ReLU” operation, is introduced.

**FIGURE 5:**
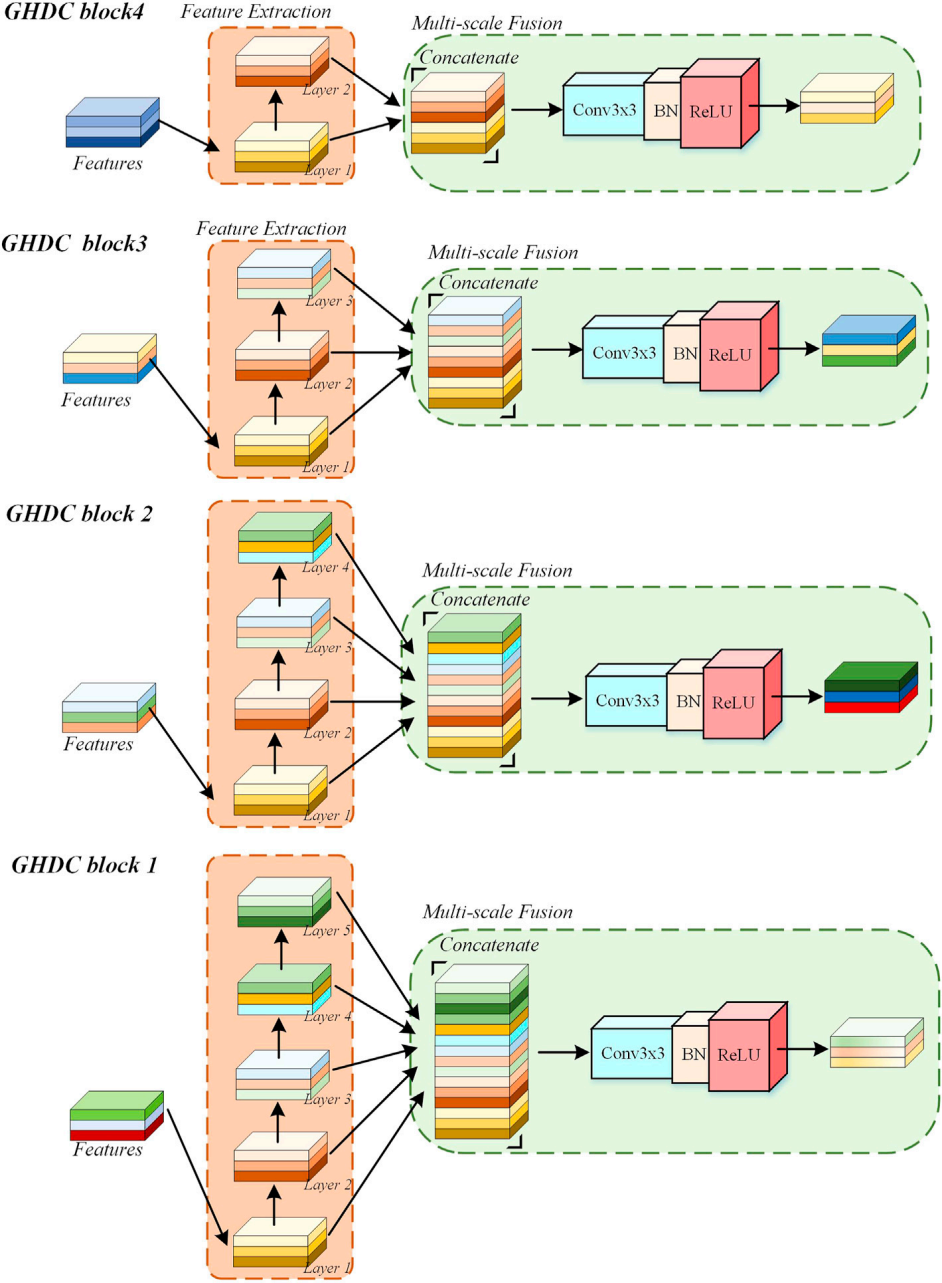
The detailed structure of the GHDC block.

In Table 1, the dilation rates and the convolutional kernel sizes of the dilated convolutional layers, as well as the receptive field sizes, are provided for the four GHDC blocks. The convolutional kernel sizes are odd. Thus, unique and consistent central pixel locations, as well as symmetric padding operations, can be guaranteed during the convolutions. Meanwhile, integer values of *m*_*l*,*i*_ and *n*_*l*,*i*_ in Eq. 1 are ensured. The detailed structures of the four GHDC blocks are shown in Figure 5.

**TABLE 1:** The arguments for the GHDC blocks in the proposed N-Net.

GHDC blocks	The number of Layers *i* *i* = 1, 2, 3, 4, 5	Dilation Rate [*r*_1_, *r*_2_, … ,*r*_*i*_]	Kernel Size [*k*_1_, *k*_2_ … ,*k*_*i*_]	Respective Fields
GHDC block4	*i* = 2	[1,2]	[3,3]	7
GHDC block3	*i* = 3	[1,2,5]	[3,3,3]	17
GHDC block2	*i* = 4	[1,2,5,7]	[3,3,3,5]	45
GHDC block1	*i* = 5	[1,2,5,7,9]	[3,3,3,5,5]	81

## 3 Experiments and results

### 3.1 Dataset

In order to verify the proposed N-Net, two publicly available datasets on polyp segmentations of colonoscopy images were used in the experiments: 1) the Kvasir-SEG dataset (Jha et al., 2019); 2) the CVC-ClinicDB dataset (Bernal et al., 2015). Both the original images and corresponding masks for the ground truth of the lesion regions are included in the two datasets. The details of these two datasets are provided in Table 2. The images from the Kvasir-SEG dataset and the CVC-ClinicDB dataset were resized into 256 × 320 and 288 × 384, respectively, before used in the experiments. We randomly divided the datasets into the training sets, the test set and the validation set, according to the ratio of 7:2:1.

**TABLE 2:** The two publicly available colorectal polyp segmentations datasets used in experiments.

Dataset	Images	Train	Validation	Test
Kvasir-SEG	1,000	715	104	189
CVC-ClinicDB	612	429	60	123

### 3.2 Implementation details

The proposed N-Net was trained based on the gradient descent method. The training process was performed with the Python package PyTorch 1.11.0 + GPU, using a computer with the Nvidia GTX 3090 GPU, and the RAM size of 32.00 GB. We used the Adaptive Moment Estimation (Adam) (Kingma and Ba, 2017) to control the learning rates in the training process, with the initial learning rate, the weight decay, the batch size and the number of epochs as 10^–4^, 10^–8^, 16 and 300, respectively. The sum of the binary cross-entropy and the soft dice coefficient (Sun et al., 2016) was used as the loss function in the training process of the N-Net. The binary cross-entropy and the soft dice coefficient are defined as:

$$L_{bce}=-\overset{n}{\underset{i=1}{\sum }}\left[{\hat{y}}_{i0}{lny}_{i0}+\left(1-{\hat{y}}_{i0}\right)\ln\left(1-y_{i0}\right)\right]$$ (5)

and

$$L_{\mathrm{dice}}=1-\frac{2\overset{n}{\underset{i=1}{\sum }}y_{i1}{ŷ}_{i1}}{\overset{n}{\underset{i=1}{\sum }}y_{i1}+\overset{n}{\underset{i=1}{\sum }}{ŷ}_{i1}},$$ (6)

respectively.

In Eqs 5, 6, *n* denotes the number of samples in a training data batch. *y*_*im*_ and *y*_*im*_ represent the predicted and actual probabilities that the *ith* pixel belongs to the class *m* (*m* ∈ {0, 1}), respectively.

To verify the performance of the proposed method, the metrics of Dice, ASSD and mIoU were implemented on the lesion region segmentation results in the experiments.

The metric of Dice is defined as:

$$Dice=\frac{2|S\cap G|}{\left|S|+|G\right|},$$ (7)

where *S* and *G* represent the region segmented by the N-Net and in the ground truth, respectively.

The metric of ASSD is defined as:

$$ASSD=\frac{\underset{m\in P_{a}}{\sum }d\left(m,P_{b}\right)+\underset{n\in P_{b}}{\sum }d\left(n,P_{a}\right)}{\left|P_{a}|+|P_{b}\right|},$$ (8)

where *P*_*a*_ and *P*_*b*_ denote the set of boundary points segmented by the convolutional neural network (CNN) and in the ground truth, respectively. $d\left(u,P_{b}\right)=\underset{v\in P_{b}}{\min}\|u-v\|$ represents the minimum Euclidean distance from the point *u* to *P*_*b*_.

The metric of mIoU is defined as:

$$mIoU=\frac{1}{2}\left(\frac{TP}{FN+FP+TP}+\frac{TN}{FP+FN+TN}\right),$$ (9)

where *TP*, FP, *TN* and *FN* represent the numbers of pixels with true positive, false positive, true negative and false negative decisions for the lesion regions, respectively.

### 3.3 Results

#### 3.3.1 Ablation studies on the proposed GHDC blocks of the N-Net

In order to verify the effectiveness of the N-Net structure and analyze the contributions of the GHDC blocks to the network performance, we first performed the ablation experiments with the GHDC blocks on the two datasets. The results of the experiments are shown in Table 3. In that table, the U-Net structure with the encoding path transferred from the first four stages of the DenseNet-121 is denoted as the baseline. By observing the results from the networks with only a single GHDC block included, we can find that the GHDC blocks greatly help in the segmentation performance improvement in terms of the Dice, ASSD and mIoU values. In particularly, this contribution increases sequentially from the GHDC block four to the GHDC block 1. Moreover, it can also be observed that by integrating more GHDC blocks into the network structures, the segmentation performances of the resulting networks can be further improved, as the multi-scale information can be transmitted more sufficiently in these cases. At the end of Table 3, we can see that the N-Net, where all of the four GHDC blocks are included, achieve the best lesion region segmentation performance on both of the two datasets.

**TABLE 3:** Ablation Study Results of the GHDC Blocks on the Two Public Datasets of Colorectal Polyp Segmentations (The best results and the second best results are marked in red bold and blue bold fonts, respectively).

Results on the Kvasir-SEG Dataset.				
Baseline	GHDC block *i*	Dice (%)	ASSD (pix)	mIoU (%)
*√*	×	92.06 (±4.74)	0.51 (±0.20)	85.92 (±4.63)
*√*	*i* = 4	92.23 (±5.02)	0.49 (±0.43)	86.08 (±7.14)
*√*	*i* = 3	92.26 (±3.64)	0.49 (±0.70)	86.19 (±3.63)
*√*	*i* = 2	92.43 (±4.31)	0.48 (±0.67)	86.34 (±4.69)
*√*	*i* = 1	92.97 (±3.86)	0.46 (±0.42)	86.60 (±5.53)
*√*	*i* = 4, 3	92.53 (±3.05)	0.46 (±0.36)	86.48 (±4.00)
*√*	*i* = 4, 2	92.70 (±3.66)	0.45 (±0.47)	86.61 ± 5.26)
*√*	*i* = 4, 1	93.15 (±4.28)	0.44 (±0.53)	86.83 (±4.37)
*√*	*i* = 3, 2	92.62 (±4.35)	0.47 (±0.49)	86.56 (±6.01)
*√*	*i* = 3, 1	93.24 (±7.68)	0.43 (±0.35)	87.07 (±4.49)
*√*	*i* = 2, 1	93.53 (±3.66)	0.42 (±0.17)	87.24 (±4.03)
*√*	*i* = 4, 3, 2	93.84 (±5.30)	0.40 (±0.20)	87.93 (±7.03)
*√*	*i* = 3, 2, 1	**94.22(** **±** **3.57)**	**0.41(** **±** **0.26)**	**89.42(** **±** **3.80)**
	**N-Net**	**94.45(** **±** **1.48)**	**0.38(** **±** **0.21)**	**89.80(** **±** **2.56)**
**Results on the CVC-ClinicDB Dataset.**				
**Baseline**	**GHDC block *i***	**Dice (%)**	**ASSD (pix)**	**mIoU (%)**
*√*	×	95.15 (±2.01)	0.32 (±0.40)	90.63 (±2.96)
*√*	*i* = 4	95.20 (±2.52)	0.30 (±0.31)	90.92 (±4.30)
*√*	*i* = 3	95.41 (±1.94)	0.29 (±0.29)	91.04 (±5.37)
*√*	*i* = 2	95.83 (±2.42)	0.27 (±0.33)	91.16 (±6.61)
*√*	*i* = 1	96.02 (±5.28)	0.26 (±0.27)	91.32 (±4.72)
*√*	*i* = 4, 3	95.49 (±5.45)	0.28 (±0.43)	91.20 (±6.20)
*√*	*i* = 4, 2	95.98 (±3.26)	0.26 (±0.28)	91.37 ± 3.65)
*√*	*i* = 4, 1	96.13 (±6.20)	0.24 (±0.19)	91.73 (±4.01)
*√*	*i* = 3, 2	96.05 (±6.07)	0.25 (±0.20)	92.09 (±3.84)
*√*	*i* = 3, 1	96.29 (±5.32)	0.23 (±0.39)	92.56 (±5.28)
*√*	*i* = 2, 1	96.46 (±5.00)	0.21 (±0.09)	92.89 (±6.25)
*√*	*i* = 4, 3, 2	96.62 (±7.08)	0.19 (±0.10)	92.01 (±2.02)
*√*	*i* = 3, 2, 1	**96.91(** **±** **5.34)**	**0.18(** **±** **0.21)**	**94.13(** **±** **4.60)**
	**N-Net**	**97.03(** **±** **0.82)**	**0.16(** **±** **0.06)**	**94.35(** **±** **1.49)**

Besides, we also conducted experiments to verify the advantage of the GHDC blocks compared the HDC on the two public colorectal polyp segmentation datasets. In particular, we compared the networks integrating the GHDC block 1 and/or the GHDC block 2 with the cases where the corresponding GHDC block(s) were/was replaced with the HDC(s). The HDCs in the experiments were implemented as proposed in (Wang et al., 2018) (i.e. the maximum number of cascaded convolutional layers was *n* = 4; the dilation rates and convolutional kernel sizes of the layers were taken as *R*_1_ = 1, *R*_2_ = 2, *R*_3_ = 5, *R*_4_ = 9 and *k*_1_ = *k*_2_ = *k*_3_ = *k*_4_ = 3, respectively). The experimental results are shown in Table 4.

**TABLE 4:** Experimental Results of GHDC and HDC Implementations Based on the Two Public Datasets of Colorectal Polyp Segmentations (The best results are marked in red bold font).

Results on the Kvasir-SEG Dataset						
Network	Dilated Convolution Settings		RF	Dice (%)	ASSD (pix)	mIoU (%)
	Dilation Rate	Kernel Size				
Baseline	\	\	\	92.06 (±4.74)	0.51 (±0.20)	85.92 (±4.63)
Baseline + GHDC 2	**[1,2,5,7]**	**[3,3,3,5]**	**45**	**92.43(** **±** **4.31)**	**0.48(** **±** **0.67)**	**86.34(** **±** **4.69)**
Baseline + HDC	[1,2,5,9]	[3,3,3,3]	35	92.25 (±3.14)	0.49 (±0.50)	86.31 (±5.23)
Baseline + GHDC 1	**[1,2,5,7,9]**	**[3,3,3,5,5]**	**81**	**92.97(** **±** **3.86)**	**0.46(** **±** **0.42)**	**86.60(** **±** **5.53)**
Baseline + HDC	[1,2,5,9]	[3,3,3,3]	35	92.54 (±4.30)	0.48 (±0.22)	86.59 (±6.02)
Baseline + GHDC 2 + GHDC 1	−	−	−	**93.53(** **±** **3.66)**	**0.42(** **±** **0.17)**	**87.24(** **±** **4.03)**
Baseline + HDC + HDC	−	−	−	92.97 (±4.51)	0.45 (±0.29)	86.91 (±4.60)

**Results on the CVC-ClinicDB Dataset**						
**Network**	**Dilated Convolution Settings**		**RF**	**Dice (%)**	**ASSD (pix)**	**mIoU (%)**
	**Dilation Rate**	**Kernel Size**				
Baseline	\	\	\	95.15 (±2.01)	0.32 (±0.40)	90.63 (±2.96)
Baseline + GHDC 2	**[1,2,5,7]**	**[3,3,3,5]**	**45**	**95.83(** **±** **2.42)**	**0.27(** **±** **0.33)**	**91.16(** **±** **6.61)**
Baseline + HDC	[1,2,5,9]	[3,3,3,3]	35	95.71 (±3.30)	0.28 (±0.44)	91.09 (±8.30)
Baseline + GHDC 1	**[1,2,5,7,9]**	**[3,3,3,5,5]**	**81**	**96.02(** **±** **5.28)**	**0.26(** **±** **0.27)**	**91.32(** **±** **4.72)**
Baseline + HDC	[1,2,5,9]	[3,3,3,3]	35	95.94 (±6.01)	0.27 (±0.45)	91.24 (±6.66)
Baseline + GHDC 2 + GHDC 1	−	−	−	**96.46(** **±** **5.00)**	**0.21(** **±** **0.09)**	**92.89(** **±** **6.25)**
Baseline + HDC + HDC	−	−	−	96.17 (±4.68)	0.22 (±0.34)	92.79 (±7.05)

From Table 4, we can observe that the networks integrating the GHDC blocks outperform the corresponding networks using the HDC on both of the two datasets, in terms of Dice, ASSD and mIoU values. As the receptive fields of both the GHDC block two and the GHDC block 1 are larger than that of the HDC, especially the receptive field generated by the GHDC block 1 is even more than twice that of the HDC, the proposed GHDC blocks are able to exploit more features, resulting in more powerful networks.

#### 3.3.2 Visual inspection of the feature maps obtained by GHDC blocks

**FIGURE 6:**
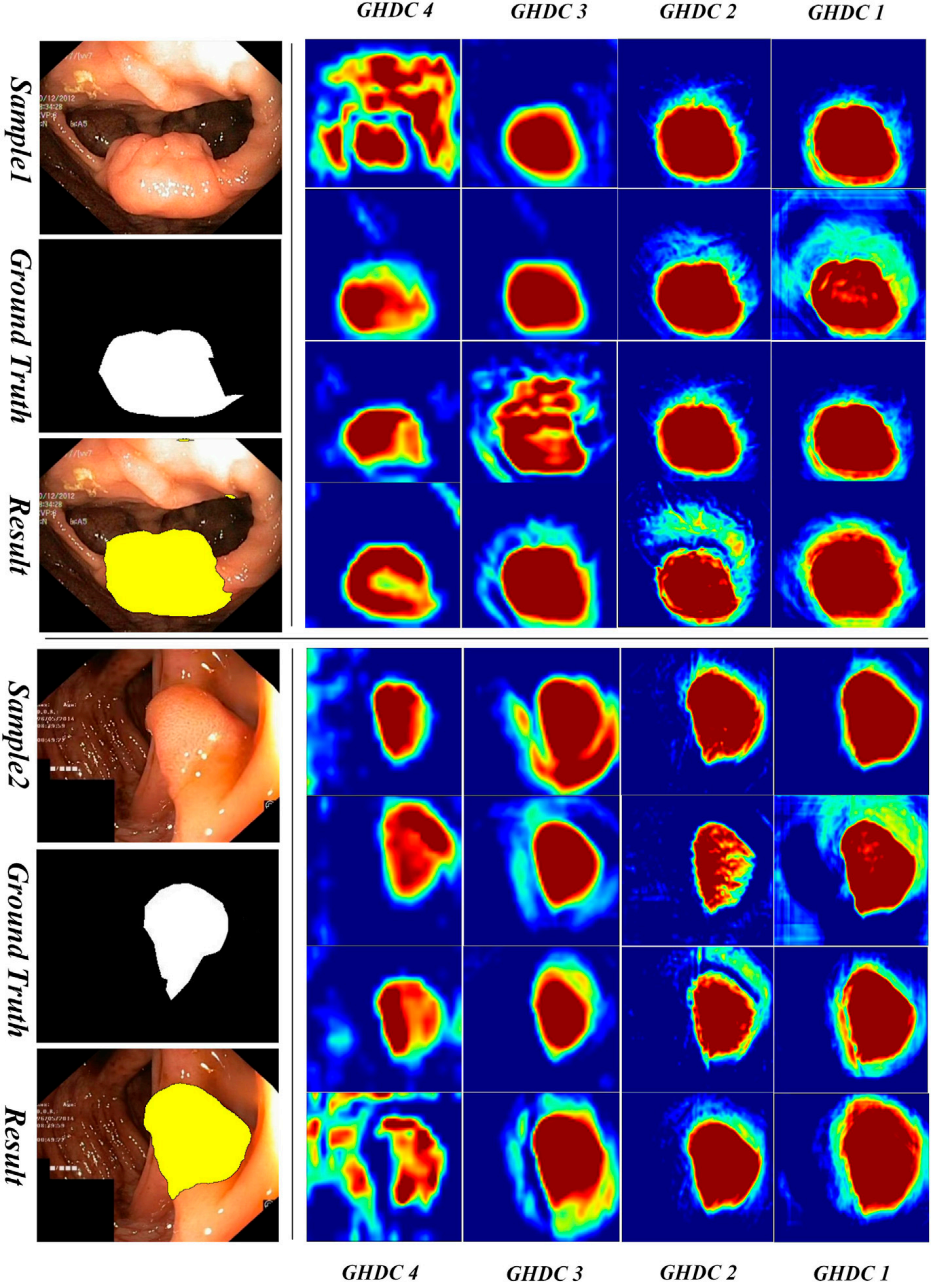
Visualization of the feature maps from the GHDC blocks on the Kvasir-SEG dataset.

In order to obtain a deeper understanding of the GHDC benefits, we analyzed the feature maps from the proposed GHDC blocks by visual inspection. In the proposed N-Net, the feature maps from the GHDC blocks 1, 2, 3 and 4 contain 64, 256, 512 and 1,024 channels, respectively. As The channels from the feature map of a GHDC block get similar features, four channels are randomly picked from the feature map from each GHDC block as representative channels. Results from two representative colonoscopy images in the testing set are shown in Figure 6, 7 for each of the two publicly available datasets, respectively.

**FIGURE 7:**
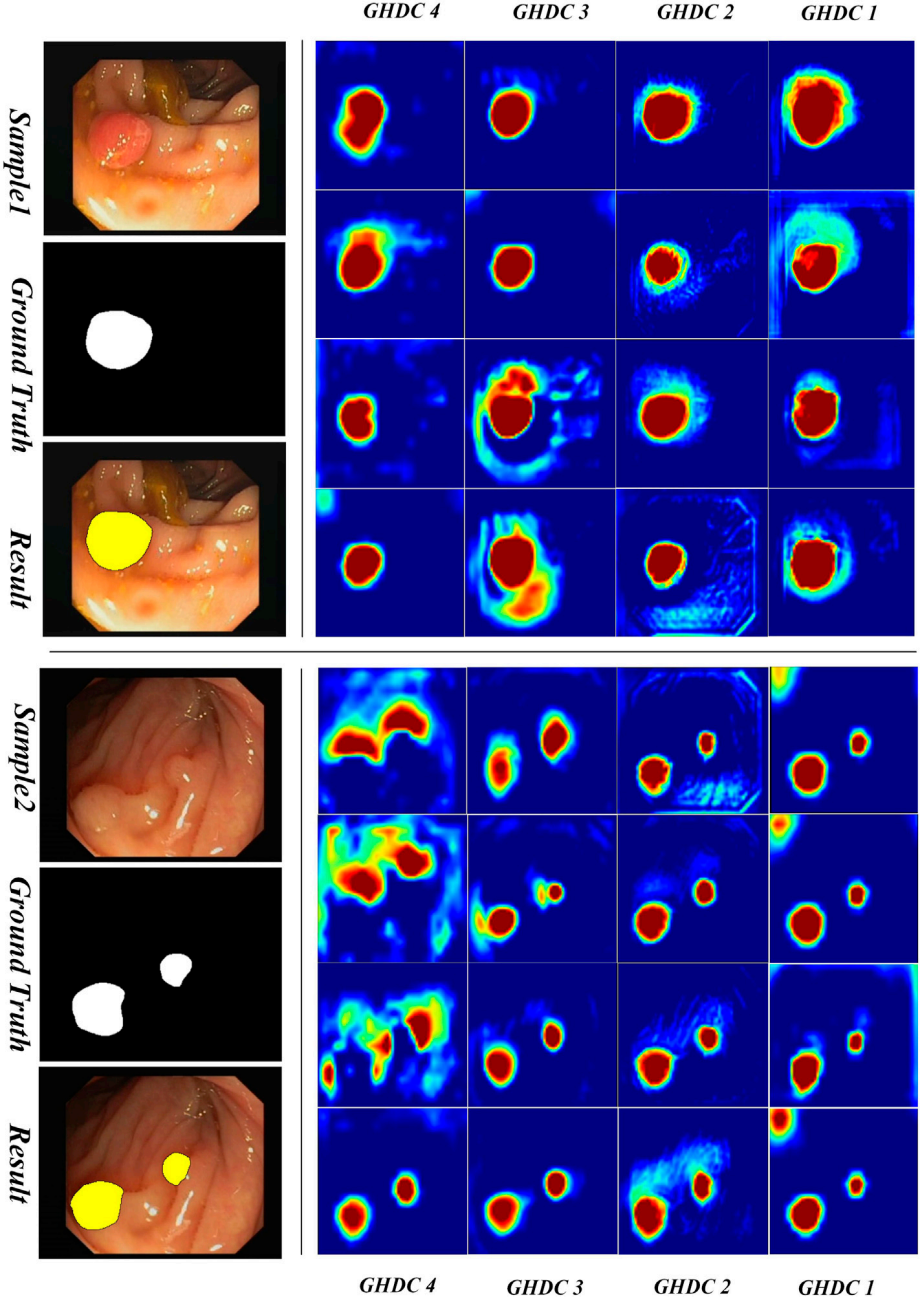
Visualization of the feature maps from the GHDC blocks on the CVC-ClinicDB dataset.

From Figures 6, 7 we can observe that the feature map extracted by the GHDC blocks gets gradually richer details from the GHDC block 4 to the GHDC block 1. Compared to the GHDC blocks 3 and 4, the GHDC blocks 1 and 2 are able to capture more fine-grained features and generate feature maps with higher resolutions. Moreover, it is worth mentioning that the feature maps from all of the 4 GHDC blocks (not only the GHDC blocks 1 and 2) are highly correlated to the final segmentation results. This in turn illustrates that the semantic information exploited by each stage of the proposed N-Net is sufficiently utilized and that the transmission of multi-scale information is facilitated with the proposed GHDC blocks.

#### 3.3.3 Comparative studies between the proposed N-Net and the state-of-the-art methods

Finally, we conducted comparative lesion region segmentation studies between the proposed N-Net and the state-of-the-art methods to verify the advantage of the proposed method. The experiments were conducted on the above publicly available polyp segmentation datasets on colonoscopy images. A variety of the state-of-the-art methods, including the U-Net (Ronneberger et al., 2015), the DeepLabV3+ (Chen et al., 2018), the U-Net++ (Zhou Z. et al., 2018), the U-Net+++ (Huang et al., 2020), Attention U-Net (Oktay et al., 2018), TransU-Net (Chen et al., 2021),OCR-Net (Wang et al., 2021) and CA-Net (Gu et al., 2021) were selected for comparison. The experimental results are shown in Table 5.

**TABLE 5:** Performance Comparison of the proposed N-Net to the State-of-the-art Methods on the Two Public Datasets of Colorectal Polyp Segmentations.

Results on the Kvasir-SEG Dataset.				
**Network**	**Parameter Size**	**Dice (%)**	**ASSD (pix)**	**mIoU (%)**
U-Net	39.4** **M	87.10 (±1.84)	0.82 (±0.15)	78.41 (±2.71)
U-Net++	47.2** **M	88.16 (±3.72)	0.78 (±0.18)	80.12 (±5.40)
Attention U-Net	34.5** **M	88.60 (±2.37)	0.81 (±0.31)	80.60 (±3.39)
U-Net+++	27.0** **M	89.20 (±3.01)	0.73 (±0.30)	81.51 (±4.65)
CA-Net	44.4** **M	89.58 (±3.40)	0.71 (±0.44)	82.17 (±5.19)
TransU-Net	133.4** **M	91.84 (±3.44)	0.52 (±0.84)	85.71 (±5.41)
OCR-Net	70.4** **M	92.24 (±2.85)	0.46 (±0.47)	86.26 (±4.52)
DeeplabV3+	39.6** **M	**94.07(** **±** **1.73)**	**0.39(** **±** **0.22)**	**89.08(** **±** **2.85)**
**N-Net**	**20.6 M**	**94.45(** **±** **1.48)**	**0.38(** **±** **0.21)**	**89.80(** **±** **2.56)**
**Results on the CVC-ClinicDB Dataset.**				
**Network**	**Parameter Size**	**Dice (%)**	**ASSD (pix)**	**mIoU (%)**
U-Net	39.4** **M	91.58 (±1.99)	0.68 (±0.16)	85.29 (±3.04)
U-Net++	47.2** **M	91.64 (±1.63)	0.71 (±0.25)	85.78 (±2.44)
Attention U-Net	34.5** **M	93.53 (±1.78)	0.70 (±0.28)	88.39 (±2.88)
U-Net+++	27.0** **M	94.70 (±1.31)	0.47 (±0.28)	90.30 (±2.16)
CA-Net	44.4** **M	94.58 (±2.51)	0.47 (±0.43)	90.20 (±4.15)
TransU-Net	133.4** **M	94.98 (±1.43)	0.33 (±0.15)	90.27 (±2.35)
OCR-Net	70.4** **M	95.35 (±1.44)	0.39 (±0.19)	91.42 (±2.46)
DeeplabV3+	39.6** **M	**96.87(** **±** **0.75)**	**0.19(** **±** **0.06)**	**94.14(** **±** **1.36)**
**N-Net**	**20.6 M**	**97.03(** **±** **0.82)**	**0.16(** **±** **0.06)**	**94.35(** **±** **1.49)**

Through the segmentation performance comparisons on the Kvasir-SEG dataset, it is important to mention that the N-Net can guarantee the best segmentation performance with the parameter amount minimized. The reduction of the parameters in turn greatly improves the efficiency of the algorithm. From Table 5, we can also observe that the performance of DeepLabV3+ is the closest to the N-Net with the above three metrics. However, the parameter size of 39.6 M is needed for the DeepLabV3+, while only 20.6 M is needed for the N-Net. Compared with the N-Net, the metrics of Dice, ASSD and mIoU for the U-Net+++ gets worse by 5.25%, 0.35 pix and 8.29%, respectively, though the size of its parameters is increased by 6.4 M.

From Table 5, we can also observe that the N-Net shows the best segmentation performance on the CVC-ClinicDB dataset with the metrics of Dice, ASSD and mIoU reaching 97.03%, 0.16 pix, and 94.35%, respectively, with the smallest computational parameter amount.

**FIGURE 8:**
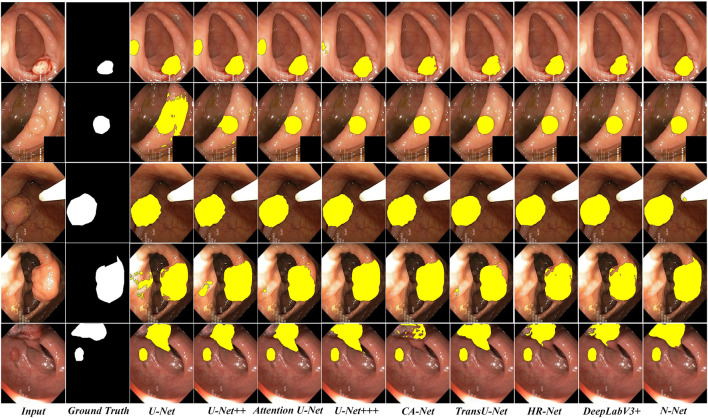
Segmentation results of the N-Net and the state-of-the-art methods on representative images of the Kvasir-SEG dataset.

**FIGURE 9:**
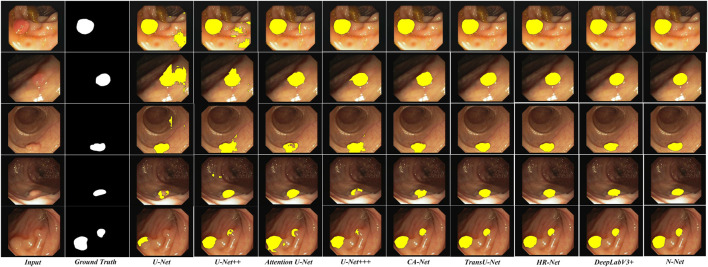
Segmentation results of the N-Net and the state-of-the-art methods on representative images of the CVC-ClinicDB dataset.

To be more intuitive, we also compared the lesion region segmentation results of the above networks. Some representative comparison results are shown in Figures 8, 9, respectively. The segmentation results from the colonoscopy images are marked in yellow. From those figures, we observe that the segmentation results with the proposed N-Net method is the closest to the ground truth. Moreover, the isolated false predictions by the proposed N-Net are also minimized. This is because the proposed N-Net is able to utilize the multi-scale features sufficiently, with the receptive fields ensured during the multi-scale information transmissions.

## 4 Discussion

In this paper, we propose a new N-shaped deep neural network (N-Net) structure to conduct the lesion region segmentations of the colorectal polyps from colonoscopy images. To facilitate the multi-scale information transmissions, we propose a strategy of generalized hybrid dilated convolution (GHDC) designing which enables flexible dilation rates and convolutional kernel sizes to facilitate transmission of the multi-scale information. Based on the proposed strategy of GHDC designing, we design four GHDC blocks to connect the encoding path and the decoding path of the N-Net.

The proposed method was evaluated on two publicly available colonoscopy image datasets for polyp segmentations: the Kvasir-SEG dataset and the CVC-ClinicDB dataset. The advantages of the proposed GHDC blocks were demonstrated through multiple sets of ablation experiments. In addition, the interpretability of the proposed GHDC blocks was analyzed through the visualization of the feature maps. Moreover, through comparative studies, the proposed N-Net was shown to outperform the state-of-the-art CNNs, including DeepLabV3+, TransU-Net and CA-Net, with the metrics of Dice, ASSD and mIoU as 94.45%, 0.38 pix and 89.80% on the Kvasir-SEG dataset and 97.03%, 0.16 pix and 94.35% on the CVC-ClinicDB dataset, respectively.

In this paper, the research was conducted on two publicly available datasets of polyp segmentations for colonoscopy images, the Kvasir-SEG dataset and the CVC-ClinicDB dataset, where consistency were shown in the results. As the patient amount included in the two datasets was still limited, additional datasets (Vázquez et al., 2017; Misawa et al., 2020; Sánchez-Peralta et al., 2020; Li et al., 2021; PIBAdb, 2022) can also be considered in the algorithm development and validations in our future work. In addition, we will also explore to implement the proposed GHDC theory and the designed blocks in other biomedical image segmentation tasks in our future work.

## 5 Conclusion

In this work, we proposed an N-Net structure based on the encoding-decoding structure to conduct the polyp lesion region segmentations of colonoscopy images. In the proposed N-Net, the pre-trained DenseNet module was transferred as the encoding path of the network. In particular, we proposed a strategy of generalized hybrid dilated convolution (GHDC) designing to facilitate transmission of the multi-scale information and expand the respective fields. Based on the strategy of GHDC designing, four GHDC blocks were designed to connect the encoding path and the decoding path of the N-Net. Experiments were performed on two publicly available colorectal polyp lesion region segmentation dataset: the Kvasir-SEG dataset and the CVC-ClinicDB dataset. The advantages of the GHDC blocks were verified. Moreover, experimental results also showed that the proposed N-Net outperforms with a small amount of parameters, compared with the state-of-the-art methods.

## Data Availability

Two publicly available polyp segmentation datasets of the colonoscopy images (the Kvasir-SEG dataset and the CVC-ClinicDB dataset) were used in the experiments of this study. The Kvasir-SEG dataset can be found at: https://datasets.simula.no/kvasir-seg/. The CVC-ClinicDB dataset can be found at: https://www.kaggle.com/datasets/balraj98/cvcclinicdb.
